# 2‐(N‐Hexylcarbazole‐3′‐yl)‐4‐pyridinealdehyde: Cyanide Detection via Benzoin Condensation

**DOI:** 10.1002/bio.70274

**Published:** 2025-08-01

**Authors:** Ahmet Battal, Mustafa Tavasli

**Affiliations:** ^1^ Department of Elementary School of Education Faculty of Education, Muş Alparslan University Muş Türkiye; ^2^ Department of Chemistry Faculty of Science‐Art, Bursa Uludag University Nilufer, Bursa Türkiye

**Keywords:** 1,2‐nucleophilic addition, benzoin condensation, carbazole, cyanide, ratiometric fluorescent sensor, real‐world samples

## Abstract

In this research, 2‐(N‐hexylcarbazole‐3′‐yl)‐4‐pyridinealdehyde (probe **A**) with a donor‐pi‐acceptor (D‐π‐A) structure was tested as a new fluorescence sensor. The fluorescence sensor properties of probe **A** were investigated by using UV–Vis and PL spectrophotometers. The absorption spectrum of probe **A** did not show a significant change against analytes added to the solution. However, when the emission spectrum of probe **A** was examined, it gave two very low‐intensity emissions at 400 nm and 540 nm. Probe **A** showed superior selectivity only for cyanide ions (CN^−^) with the limit of detection (LOD) of 1.42 nM in the presence of various competing analytes. This LOD value is the lowest value reported so far. For the first time, CN^−^ sensing proceeds via benzoin condensation reaction. The stoichiometric ratio and detection mechanism between probe **A** and CN^−^ were confirmed by Job's plot, HRMS, ^1^H‐NMR, and FT‐IR analyses. Probe **A** also worked successfully in the detection of CN^−^, which is a highly hazardous and toxic substance, including practical applications (three different real‐world water samples). Therefore, probe **A** was introduced to the field as an effective and striking potential sensor candidate in water quality testing and disease diagnosis, etc.

## Graphical abstract

Probe **A** with carbazole‐pyridine‐aldehyde (D‐π‐A) was designed as a new ratiometric fluorescence sensor. Probe **A** showed highly selectivity and sensitivity towards CN^−^ over the other competing anions, cations and neutral molecules. Surprisingly, the CN^−^ sensing mechanism proceeded via benzoin condensation reaction. The LOD value was obtained as 1.42 nM which is the smallest value reported until now. Concisely, probe **A** is a potential candidate for the detection of CN^−^ in liquid media (MeCN:water) as a ratiometric sensor.

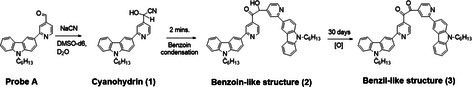

## 1 Introduction

Cyanide is an extremely dangerous and lethal substance for living things [1]. It is naturally found in many foods [2] such as seeds of ginkgo and apples, pears, apricots, prunes, cassavas and sprouting potatoes, cherries, plums, lima beans, and almonds, and it can also be formed during some chemical interactions [3]. It can also mix into drinking water as waste in many industrial chemical processes [4]. Even while the United States Environmental Protection Agency has announced a limit of 7.8 μM, the World Health Organization (WHO) has declared CN^−^ level in drinkable waters beyond a certain amount (1.9 μM) as dangerous [5]. Most importantly, it is present in cigarette smoke and fire smoke [6]. For this reason, living things, especially humans, are exposed to CN^−^ directly [7] or indirectly [8]. Therefore, in case of exposure to CN^−^, it is important to determine the amount of CN^−^ accurately, sensitively, quickly, and reliably, for certain diseases occur if indirectly and/or directly exposed to CN^−^. These diseases can range from headache, coma, vomiting, dizziness, tremors, neurodegeneration, breathlessness, arthritis, hypoxia, high heartbeats to diabetes, Alzheimer's and Parkinson's diseases, gastrointestinal tract disorders, and cancer [4, 9, 10]. To escape these diseases, it is necessary to avoid exposure to cyanide. However, to avoid the danger of life from cyanide, it is essential that its detection is carried out in the relevant areas with effective, fast, on‐site, sensitive, easy, economical, accurate, and reliable methods.

In the literature, many methods [11], such as mass spectrometry, spectroscopy, electrochemistry, ion chromatography, voltammetry, chromatography, flow injection, are used for the determination and detection of cyanide. However, due to the negative factors [12] such as the cost, the need for a laboratory environment, often time consuming, and/or requiring strict sample handling processing and complicated tools, their potential for use has decreased. However, the fluorescence sensor method has been developed to overcome such disadvantages [3, 13] in the detection of CN^−^. This technique provides advantages over the others mentioned in terms of being cheap, fast, suitable for use outside the laboratory, and requiring simple tools and equipment. Therefore, fluorescence sensor techniques have been frequently preferred in the determinations in the detection of CN^−^ [14, 15]. Fluorescence sensors that can detect low levels of cyanide are still desperately needed [16]. By us, while the lowest known detection level was 1.47 nM [11], this study broke the record, and currently, the lowest detection limit was obtained with this study.

Fluorescence sensors consist of a fluorophore unit, an analyte recognition unit (binding unit) and a linker part that connects the fluorophore and the analyte recognition unit [5]. Various materials, such as naphthalene, coumarin, quinoline, spiropyran, dibenzothiophene, phenothiazine, carbazole, are frequently used as fluorophores [17]. Generally, species such as pyridinium ring [18], oxazine [19], aldehyde [20], boronic acid [21], and dicyanovinyl [22] are also used as analyte recognition units. As linker parts, certain structures such as furan, pyrrole, thiophene, pyridine, and benzene are used [23].

Carbazole and its derivatives are materials that have been studied for many years regarding synthesis, photophysical, and fluorescence sensor properties [24]. Because they have special properties such as easy structural modification at positions 3, 6, and 9 in the structure, good intramolecular charge transfer, high stability and solubility, good hole transport, high solubility and durability, high mobility, effective electroluminescence results, high selectivity and sensitivity, low detection limit, and fast response [25]. Therefore, many studies on them are still ongoing in terms of improving their properties [26].

Fluorescence sensors work based on many reactions to detect CN^−^. These reactions can be aromatic nucleophilic substitution, nucleophilic substitution, nucleophilic addition, nucleophilic conjugated addition, electron deficient groups, H‐bonding, or ion displacement approach [11]. Among these approaches, nucleophilic addition has certain advantages over others. Due to the nucleophilic feature of CN^−^, the π‐conjugation of the structure is disrupted with the reaction between CN^−^ and the fluorophore, and the ICT feature in the structure runs out. This causes a change in the intensity/wavelength of the radiation produced by the fluorophore, which is the easiest result to see with the eye [27]. Emission mechanisms can be based on ICT‐on/off, Förster resonance energy transfer (FRET), mixed modality, excited state intramolecular proton transfer (ESIPT), photo‐induced electron transfer (PET), and so on [28]. Moreover, while the fluorescence sensors are categorized by turn‐on, turn‐off, and ratiometric. Among these, the ratiometric sensors were desired by researchers because turn‐on and turn‐off sensors can be misunderstood due to other factors (photobleaching, concentration, and environmental effects) other than the analyte. The ratiometric fluorescent sensors are clear and simple. They do not cause confusion and can be easily used in the correct determination of CN^−^ since they work measuring the ratio of fluorescence intensities of the probe at two different wavelengths [13, 29].

To obtain better detection of cyanide, which is highly toxic to living organisms, we wanted to introduce a new sensor to the literature. To the best of our knowledge, the CN^−^ sensor with the structure of carbazole‐pyridine‐aldehyde (D‐π‐A) has not been reported so far. With this respect, namely 2‐(N‐hexylcarbazole‐3′‐yl)‐4‐pyridinealdehyde (probe **A**) was selected as a new CN^−^ sensor whose synthesis, electrochemical, photophysical, solvatochromic, and biothiol sensor properties had been previously described [30, 31]. It was predicted that probe **A** would give an effective nucleophilic addition reaction with CN^−^ due to the aldehyde group present in its structure; hence, probe **A** would exhibit fluorescence sensor properties towards CN^−^. The mechanism of detection of CN^−^ by probe **A** was attributed to the nucleophilic attack of cyanide on the aldehyde (C=O) bonds by 1,2‐nucleophilic addition reaction to form a cyanohydrin structure, which quickly gave a benzoin condensation reaction. In this study, the ratiometric fluorescence sensor properties of probe **A** in acetonitrile (MeCN) towards CN^−^ were examined in the presence of various anions, cations, and neutral molecules in deionised water. The interaction mechanism of the fluorophore and analyte was determined by various analytical methods. The validity of probe **A** in practical applications was tested.

## 2 Experimental

### 2.1 Naming of Probe **A**

**FIGURE 1:**
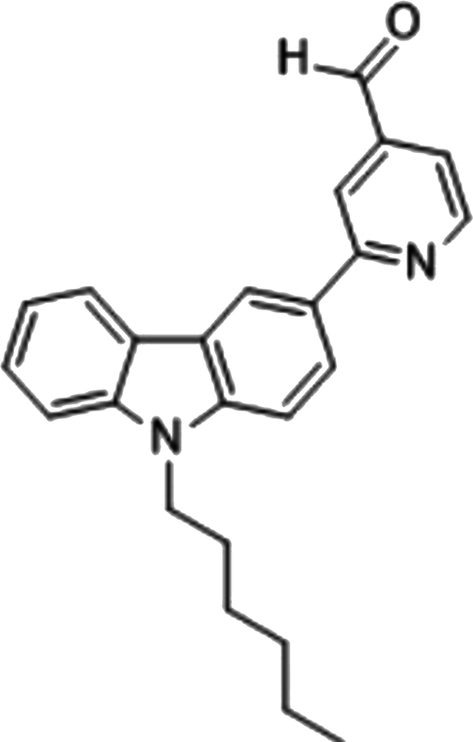
The structure of probe **A.**

2‐(N‐hexylcarbazole‐3′‐yl)‐4‐pyridinealdehyde, shown in Figure 1, was named probe **A** in this study and synthesized from carbazole in four steps as reported previously [30].

### 2.2 Chemicals and Instrumentations

To synthesize probe **A**, all the reagents purchased from different suppliers (Sigma‐Aldrich: İnterlab Istanbul, Türkiye and Arı Lab, Erzurum, Türkiye) were purely ACS reagent grade, ≥99.9% (GC), and were used without further purification. NaCN, NaF, NaCl, NaBr, KI, Na_2_SO_4_, KH_2_PO_4_, CH_3_COONa, and KNO_3_ were used for the preparation of anions, H_2_O_2_, and N_2_H_4_ were used for the preparation of neutral molecules, and FeCl_3_, FeSO_4_·7H_2_O, HgCl_2_, CuCl_2_·2H_2_O, and CoCl_2_·6H_2_O were used for the preparation of cations. All the anion and cation salts and neutral molecules used in this research were bought from the companies (Sigma‐Aldrich: Arı Lab, Erzurum, Türkiye, Chemsolute: Life Biotek, Elazığ Türkiye and Carlo Erba: Gullab, Istanbul, Türkiye) as anhydrous and granular with high purity. All aqueous solutions for these salts were prepared by using deionized water with high‐precision pipettes of different volumes. Deionized water was produced by the Milli‐Q IQ 7005 Pure & Ultrapure Water Purification System (Kartu, Istanbul, Türkiye). The Perkin Elmer Lambda 35 UV–Vis Spectrophotometer (Analytical & Enterprise Solutions, Istanbul, Türkiye) was used for the UV–Vis absorption studies. The Shimadzu RF‐5301PC Spectrofluorophotometer (Ant Teknik Company, Ankara, Türkiye) was used for the fluorescence sensor measurements. The slit width for excitation and emission measurements was 5 nm and 10 nm, respectively, at all fluorescence experiments. All absorption and emission measurements were carried out with a quartz cuvette (its light length is 10 mm and chamber volume is 3500 μL) at room temperature. The UV–Vis spectrum was recorded in the wavelength range of 250–600 nm, and the fluorescence spectrum was recorded in the wavelength range of 380–700 nm. All samples were excited at 366 nm. ^1^H‐NMR (600 MHz) spectra were recorded in DMSO‐*d6* solution at room temperature on a Bruker Ultrashield 400 MHz high‐performance nuclear magnetic resonance (NMR) spectrometer (Anatek Analitik, İstanbul, Türkiye). For this, 5.0 mg of probe **A** was well dissolved in 0.6 mL of DMSO‐*d6*, and ^1^H‐NMR was recorded. Then, 0.76 mg of NaCN in D_2_O was added to the probe **A** solution in DMSO‐*d6*, and after waiting for 2 min for incubation times, ^1^H‐NMR was recorded again. The FT‐IR spectrum of probe **A** in MeCN and probe **A**+NaCN in MeCN and deionized water at solid state and liquid state was recorded by using the ATR technique on the Agilent Cary 630 spectrometer (Altium International Lab, İstanbul, Türkiye). pH measurements of probe **A** in MeCN and probe **A** + CN^−^ in MeCN and deionized water were obtained with the Orion Star A211 Benchtop pH Meter Thermo Scientific (Ant Teknik Company, Ankara, Türkiye). The photographs of probe **A** in MeCN under a UV lamp were taken with a portable hand‐held UV lamp of 365 nm. All photographs were also taken with an iPhone 7 + smartphone. HRMS (high‐resolution mass spectra) analyses were performed at the METU Central Laboratory R&D Training and Measurement Center, Ankara, Türkiye.

### 2.3 Sample Preparation for UV–Vis and Fluorescence Studies of Probe **A**

A stock solution of probe **A** (1.0 × 10^−3^ M) in MeCN was prepared, and this stock solution was mixed stirringly for 15 min by using a Bandelin Sonorex ultrasonic cleaning bath (Kutay group, İstanbul, Türkiye). Then, by using a dilution method, 90 μL was added to a 2910 mL of the MeCN; a 3.0 × 10^−5^ M solution of probe **A** was prepared from the stock solution of probe **A** (1.0 × 10^−3^ M) by adding MeCN. Stock solutions (1.0 × 10^−2^ M) of anion salts (CN^−^, F^−^, Cl^−^, Br^−^, I^−^, SO_4_^2−^, H_2_PO_4_^−^, CH_3_COO^−^ and NO_3_^−^), neutral molecules (H_2_O_2_ and N_2_H_4_) and cations (Fe^3+^, Fe^2+^, Hg^2+^, Cu^2+^, and Co^2+^) were prepared in deionized water. After that, each time, a 30 μL solution of anions, cations, and neutral molecules was added to 2970 mL of the probe **A** solution in a quartz cuvette. The total volume was 3 mL for all tests. The interference studies of probe **A** towards CN^−^ in the presence of anions, cations, and neutral molecules were also carried out in the same way; i.e., a 30 μL solution of CN^−^ was added to the mixed solution of probe **A** and different ions. The sensitivity study was carried out by adding different concentrations of CN^−^ (0–508 μM) to a 3 mL solution of probe **A**. The mixture was incubated for 2 min at room temperature. After that, absorption (for only selectivity) and fluorescence (for all tests) were recorded. In addition, probe **A** was also used for the detection of CN^−^ in real‐world water samples, such as tap water, lake water, and river water. The tap water was collected from the Mus Municipality water system in Türkiye; river water was also collected from the Murat River of Mus province in Türkiye, and the lake water was collected from Van Lake of Van province in Türkiye. All the real‐world water samples were centrifuged at 8500 rpm for 20 min and then filtered through the membrane produced by Agilent Technologies, United States, with a pore size of 0.45 μM (19A00D184, Arı Lab, Erzurum, Türkiye) to remove the suspended particles. CN^−^ detection was carried out by the spike and recovery method, where fluorescence intensity was measured after adding different concentrations (0–140 μM, 0–200 μL) of CN^−^ into a mixture of 30 μL of real‐world water samples and 2970 μL of probe **A** in MeCN. For real‐world water experiments, the solvent ratio was obtained as 93:7 (v/v, MeCN:H₂O. The used anions, cations, and neutral molecules in this study have been selected owing to their significant relevance to real‐world samples. These ions and molecules are frequently found coexisting in various biological and environmental systems, making them potential candidates for studying sensing properties observed in the research.

## 3 Results and Discussion

### 3.1 Absorption Measurements (Colorimetric Detection)

The absorption spectrum of probe **A** in MeCN was previously measured using a Duetta Fluorescence and Absorbance Spectrometer [30]. It was seen that in the UV–Vis spectra, probe **A** displayed three specific absorption bands: high‐energy bands (285 nm) and mid‐energy bands (340 nm) were assigned to π‐π* and n‐π* transitions, whereas the low‐energy bands (366 nm) were related to an intramolecular charge transfer (ICT) transition.

**FIGURE 2:**
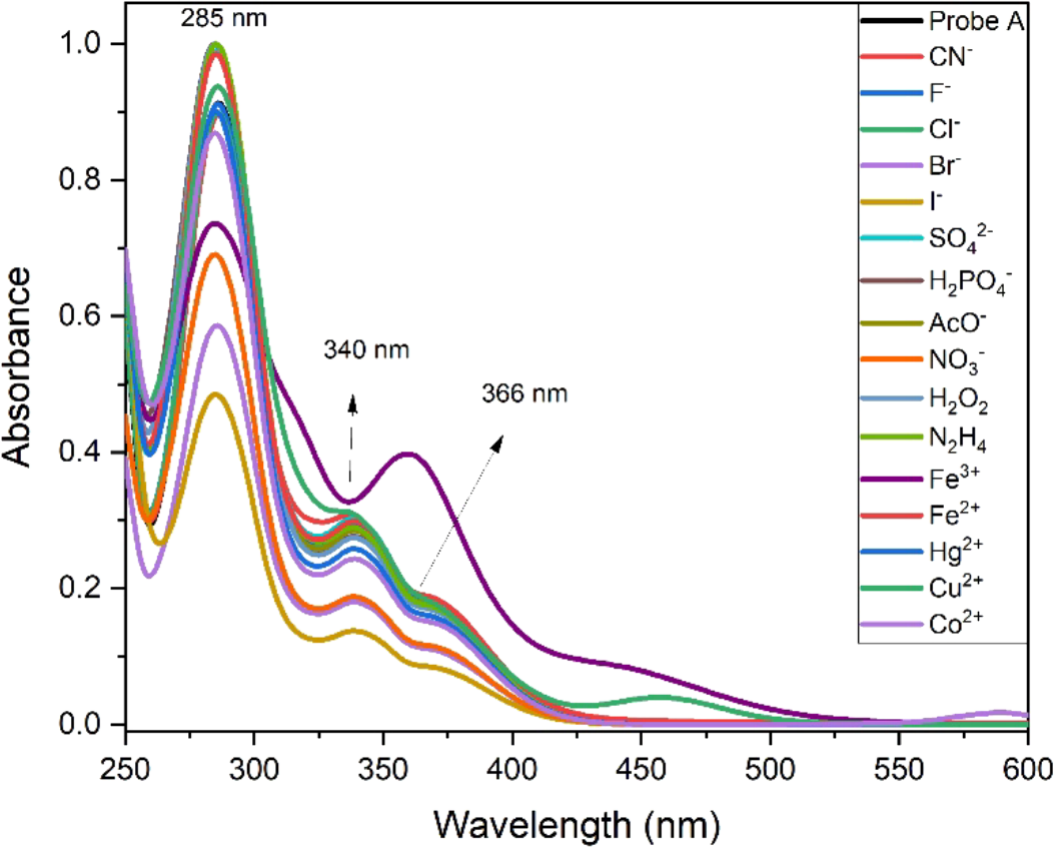
UV–Vis spectra of probe **A** (30 μM) in MeCN without analytes and with anions (CN^−^, F^−^, Cl^−^, Br^−^, I^−^, SO_4_^2−^, H_2_PO_4_^−^, CH_3_COO^−^, and NO_3_^−^), neutral molecules (H_2_O_2_ and N_2_H_4_) and cations (Fe^3+^, Fe^2+^, Hg^2+^, Co^2+^, and Cu^2+^) of 100 μM in deionized water at room temperature.

**FIGURE 3:**
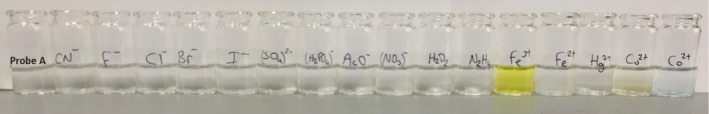
The photograph of a solution of probe **A** (30 μM) in MeCN in the presence of various anions, cations and neutral molecules (100 μM) in deionized water under daylight.

In this report, the sensing performance of probe **A** (30 μM) towards anions, cations, and neutral molecules (100 μM) was explored using Perkin Elmer Lambda 35 UV–Vis Spectrophotometer at room temperature, as shown in Figure 2. In the absorption spectrum examinations, there was no significant change by the addition of CN^−^ and other analytes in any of the existing energy bands seen in the structure, except Fe^3+^ and Cu^2+^. With the addition of Fe^3+^, the LC band slightly decreased, and the ICT band moderately increased, turning the solution color from colorless to slightly yellow (Figure 3). This change may be due to the bonding between the pyridine ring of probe **A** and Fe^3+^ or Cu^2+^.

### 3.2 Fluorometric Study (Photoluminescence (PL) Measurements)

#### 3.2.1 PL Spectra

**FIGURE 4:**
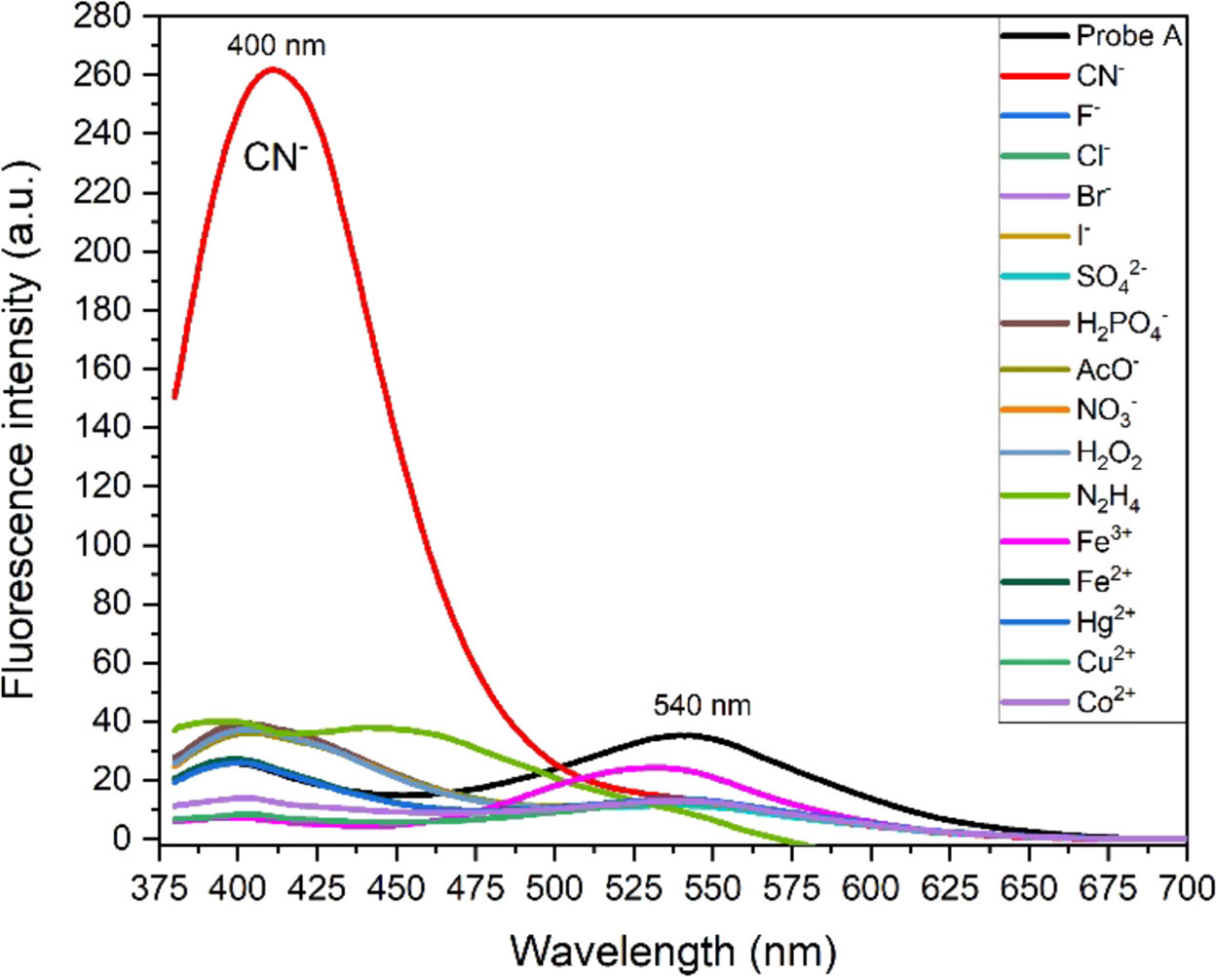
Fluorescence spectra of probe **A** (30 μM) in MeCN at room temperature without analytes and with different aqueous anions, cations and neutral molecules (100 μM) in deionized water under the excitation of 366 nm light.

The fluorescence sensor measurements of probe **A** (30 μM) were carried out in MeCN by using a Shimadzu RF‐5301PC Spectrofluorophotometer. As can be seen in the previous report [30], probe **A** had a very low relative fluorescent quantum yield (PLQY, 5.4%). Similarly, when it was excited with a 366 nm light, probe **A** gave very low emission intensity with two emission bands (see Figure 4). However, the band at 400 nm is due to ligand‐centered (LC) emission, and the band at 540 nm is due to ICT emission. The ICT emission is almost 1.36 times more intensive than the LC emission. It was evident from this result that the energy transfer from LC transition to ICT transition of probe **A** was incomplete.

#### 3.2.2 Selectivity Study of Probe **A**

In the present work, probe **A** was selected as a fluorescence probe, and it was used for the detection of anions, cations, and neutral molecules. Solutions of probe **A** (3.0 × 10^−5^ M) in MeCN and analytes (100 × 10^−6^ M) in deionized water were prepared. From various solvents, probe **A** in MeCN exhibited only a good response to CN^−^. At the addition of only water, the emission of probe **A** quenched without the presence of an analyte. Thus, all experiments were carried out in MeCN. The mixture of probe **A** and various analytes was incubated for 2 min, and then the selectivity of probe **A** was investigated at pH 7.10 and room temperature. The fluorescence spectral response of a mixture of probe **A** and various analytes was measured using a spectrofluorometer, as seen in Figure 4.

**FIGURE 5:**
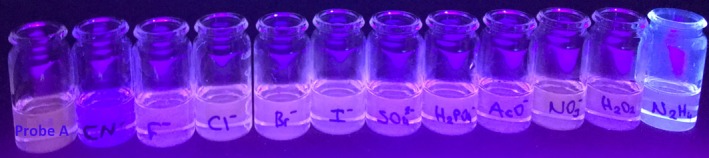
Photograph of solutions of probe **A** in MeCN, without and with anion and neutral molecules in an aqueous water medium under the UV light (365 nm).

The fluorescence intensity of probe **A** did not cause significant changes with the addition of various anions and neutral molecules examined. In addition, the ICT emission band (540 nm) of probe **A** was quenched with a slight decrease. Also, some increases in the LC emission band (400 nm) of probe **A** were observed with the addition of some anions/molecules in convenient photographs under UV light. Figure 5 shows the photograph of solutions of probe **A** in MeCN, without and with anion and neutral molecules in an aqueous water medium under the UV light at 365 nm.

**FIGURE 6:**
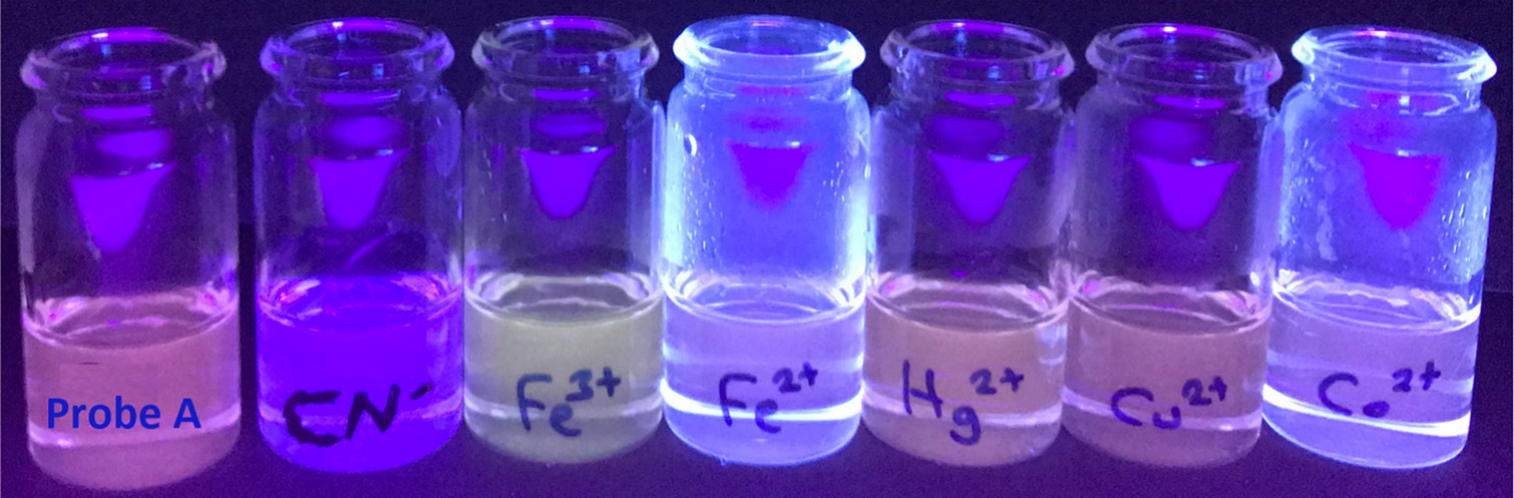
Photograph of solutions of probe **A** in MeCN, without and with CN^−^ and cations in aqueous water medium under UV light (365 nm).

The fluorescence intensity of probe **A** in MeCN was not changed much with the presence of various cations. However, the ICT emission band of probe **A** was slightly decreased for some addition of cations. Also, some increases in the LC emission band (400 nm) of probe **A** appeared with the addition of some cations in agreement with the photographs under UV light. Figure 6. shows the photograph of probe **A** in MeCN, without and with various cations in aqueous under the UV light at 365 nm.

Upon the addition of CN^−^, the LC emission band of probe **A** significantly increased while the ICT emission band decreased. The LC emission band is almost 18 times more intensive than the ICT emission. The fluorescence intensity of probe **A** has dramatically enhanced at the LC emission band and decreased in the ICT emission when only the addition of CN^−^ was made. These results showed that probe **A** detected only CN^−^ successfully. Also, the PLQY of probe **A** was calculated to be 5.4% in the absence of CN⁻ [30] and it increased to 19% with the addition of CN⁻ under the same experimental conditions.

#### 3.2.3 Sensitivity Study of Probe **A**

**FIGURE 7:**
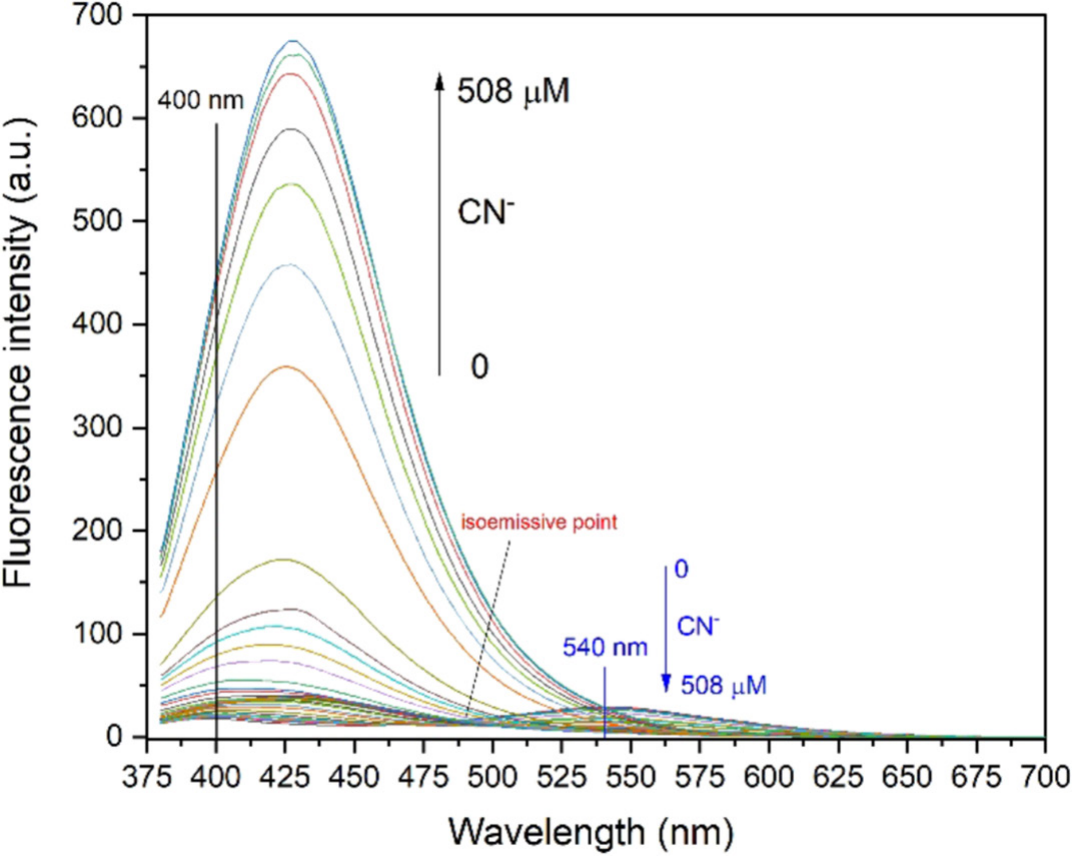
Fluorescence intensity changes of probe **A** in MeCN upon the incremental addition of CN^−^ in deionized water (0–508 μM) at room temperature (λ_exc_ = 366 nm).

To further examine the sensitivity behavior of probe **A** in MeCN towards CN^−^ in deionized water, fluorescence intensity changes of probe **A** were monitored by the incremental addition of CN^−^ (0–508 μM). For this, four different stock solutions (1.0 × 10^−3^ M, 1.0 × 10^−4^ M, 1.0 × 10^−5^ M and 1.0 × 10^−6^ M) were prepared by using the main stock solution of CN^−^ (10 × 10^−2^ M) in deionized water. As tabulated in Table 1, different volumes of [CN^−^] were taken from each of these five different solutions and then were added to the solution of probe **A** (3 mL, 3.0 × 10^−5^ M) in MeCN. The fluorescence intensity changes of probe **A** upon addition of CN^−^ were measured from 380 nm to 700 nm (Figure 7.).

**TABLE 1:** Fluorescence intensity values (at 400 nm and at 540 nm) of probe **A** in MeCN with the incremental addition of CN^−^ in deionized water.

No	[CN^−^]_initial_ (M)	Equivalent	Isoemissive point (nm)	Isoemissive point Intensity	Added CN^−^ volume (μL)	Total CN^−^ volume (μL)	[CN^−^]_final_ (μM)	Fluorescence Intensity at 400 nm	Fluorescence Intensity at 540 nm
1	1.00E‐06	—	—	—	—	—	—	18.1	29.3
2	1.00E‐06	0.00001	495 493	18	1	1	0.00033	19.1	28.1
3	1.00E‐06	0.00003	493	17	2	3	0.00100	19.4	27.9
4	1.00E‐06	0.00006	483	15	2	5	0.00166	19.8	26.1
5	1.00E‐06	0.00011	471	13	5	10	0.00332	20.4	23.1
6	1.00E‐06	0.00022	467	12	10	20	0.00662	21.6	18.9
7	1.00E‐06	0.00028	467	12	5	25	0.00826	22.5	17.7
8	1.00E‐06	0.00033	467	12	5	30	0.00990	24.0	14.8
9	1.00E‐06	0.00038	472	13	5	35	0.01153	25.6	14.3
10	1.00E‐06	0.00044	475	13	5	40	0.01316	28.2	10.8
11	1.00E‐06	0.00055	477	13	10	50	0.01639	29.7	10.0
12	1.00E‐06	0.00065	480	14	10	60	0.01961	31.1	8.8
13	1.00E‐06	0.00076	482	15	10	70	0.02280	31.0	8.2
14	1.00E‐06	0.00087	484	15	10	80	0.02597	31.7	7.6
15	1.00E‐06	0.00097	487	15	10	90	0.02913	31.3	6.9
16	1.00E‐05	0.0011	487	16	10	10	0.03226	31.6	6.7
17	1.00E‐05	0.0021	487	16	10	20	0.06431	31.7	6.5
18	1.00E‐05	0.0032	488	16	10	30	0.09615	31.6	6.5
19	1.00E‐05	0.0043	488	16	10	40	0.12780	32.4	6.4
20	1.00E‐05	0.0053	488	16	10	50	0.15924	32.4	6.4
21	1.00E‐05	0.0074	487	16	20	70	0.22152	31.4	5.7
22	1.00E‐05	0.0094	487	15	20	90	0.28302	32.2	5.3
23	1.00E‐04	0.011	487	15	10	10	0.31348	33.5	5.0
24	1.00E‐04	0.031	486	15	20	30	0.93458	34.9	4.8
25	1.00E‐04	0.052	486	15	20	50	1.54799	37.9	4.6
26	1.00E‐04	0.072	486	15	20	70	2.15385	42.7	4.6
27	1.00E‐04	0.092	487	15	20	90	2.75229	45.8	4.5
28	1.00E‐03	0.10	489	16	10	10	3.04878	53.0	4.7
29	1.00E‐03	0.30	493	17	20	30	9.09091	68.3	5.0
30	1.00E‐03	0.50	497	18	20	50	15.06024	79.5	5.5
31	1.00E‐03	0.70	500	19	20	70	20.95808	92.0	6.0
32	1.00E‐03	0.90	502	20	20	90	26.78571	101.7	6.4
33	1.00E‐02	0.99	509	22	10	10	29.67359	135.2	8.1
34	1.00E‐02	2.95	523	26	20	30	88.49558	257.4	14.2
35	1.00E‐02	4.89	528	28	20	50	146.62757	321.2	17.9
36	1.00E‐02	6.80	532	28	20	70	204.08163	368.6	21.1
37	1.00E‐02	8.70	535	29	20	90	260.86957	402.1	23.7
38	1.00E‐02	11.50	537	29	30	120	344.82759	432.8	26.3
39	1.00E‐02	14.25	539	29	30	150	427.35043	446.9	28.4
40	1.00E‐02	16.95	540	29	30	180	508.47458	440.4	29.4

While ICT emission intensity (I_540_) decreased slowly, LC emission intensity (I_400_) increased significantly with the incremental addition of CN^−^. When probe **A** was titrated with 1 equivalent of CN^−^, I_400_ increased nearly 8‐fold, while I_540_ decreased nearly 4‐fold. Careful examination of Figure 7 indicates that several isoemissive points were observed for this titration (Table 1). This might indicate the formation of different adducts between probe **A** and CN^−^.

#### 3.2.4 The Determination of Limit of Detection (LOD)

**FIGURE 8:**
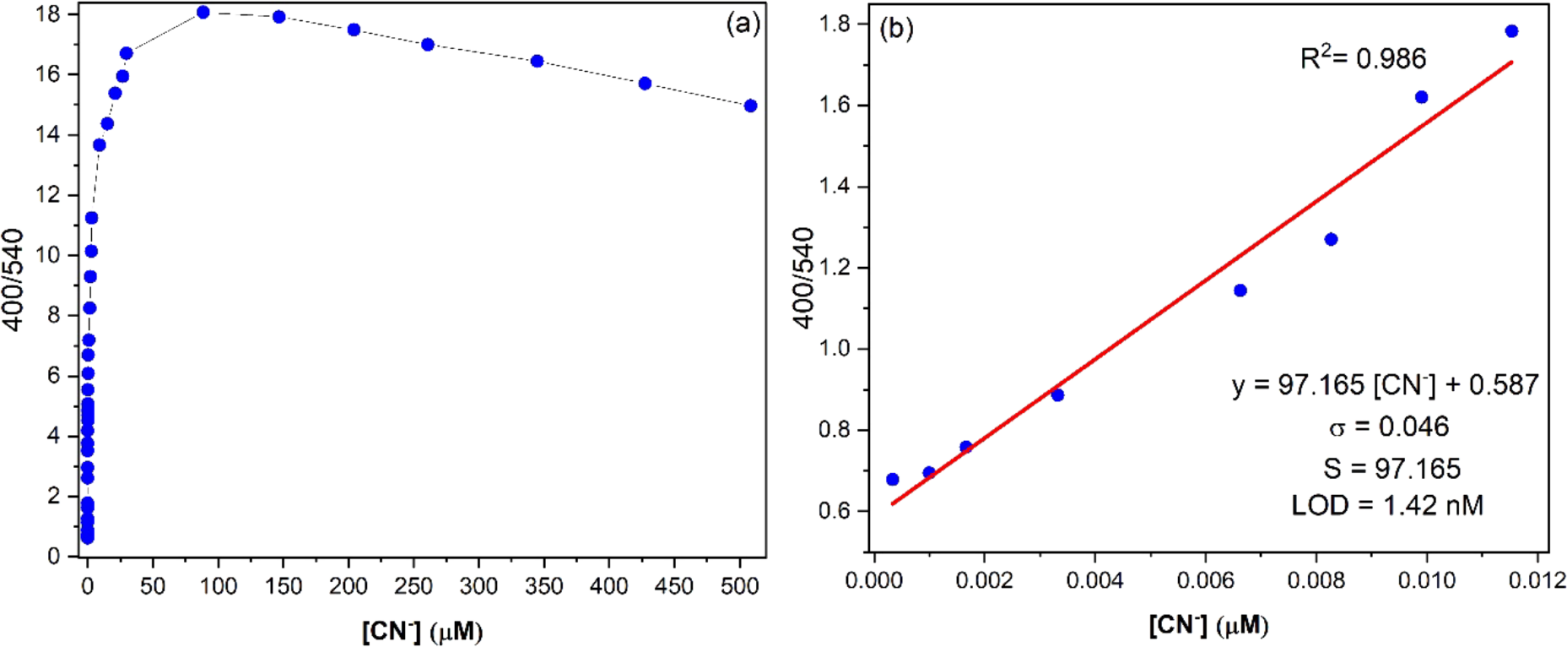
(a) The changes in I_400_/I_540_ of probe **A** vs. added [CN^−^]. (b) Linear best fit of values from the graph (a) between 0.000 and 0.012 μM with *R* ^2^ = 0.986 and its linear equation.

The LOD value of fluorescence probes can be calculated from the equation (LOD = 3σ/S), where σ is the standard deviation of the *y*‐intercept and *S* is the slope of the curve, as related to the equation of IUPAC. To calculate the S value, firstly, the I_400_/I_540_ of probe **A** against the incremental addition of CN^−^ (from 0 to 508 μM) was plotted as seen in Figure 8.

Upon the incremental addition of CN^−^, the I_400_/I_540_ ratio increases to a maximum of 18‐fold, then saturates as seen in Figure 8a. Moreover, as [CN^−^] increases from 0 to 0.012 μM, the I_400_/I_540_ ratio increases linearly with R^2^ = 0.986. This region was replotted in Figure 8b and the mathematical equation for the best linear fit is found as y = 97.165x + 0.587. From Figure 8b, σ and S values are calculated as 0.046 and 97.165, respectively. If these σ and S values are substituted into the LOD equation (3σ/S), the LOD value of probe **A** for CN^−^ detection is found to be 1.42 nM. As seen in Table 2, literature reports indicate that this LOD value (1.42 nM) is the smallest value reported so far for the detection of CN^−^. This LOD value is also lower than WHO's maximum allowed CN^−^ concentration (1.9 μM) in drinking water [39]. Therefore, probe **A** can be effectively used in the detection of CN^−^ in drinking water.

**TABLE 2:** Previously reported some fluorescent probes for CN^−^ detection and their LOD values.

Probes	Solvent system	Response time	LOD value (nM)	Year	Ref.
Benzothiazole	CH_3_CN/water	N/A	1600	2013	[32]
Quinoline	Water	N/A	58	2014	[1]
Naphthopyran‐benzothiazol	DMSO/water	N/A	290	2016	[33]
Carbazole‐cyanine dya	DMF/HEPES	5 mins	90	2019	[34]
Phenylcarbazole	THF/water	2 mins	249	2022	[35]
Diphenylamine	EtOH/water	N/A	480	2022	[36]
Carbazole‐indolium	DMSO/water	N/A	140	2024	[9]
Carbazole	DMSO	2 mins	1.47	2024	[11]
Carbazole	THF	1 min	430	2025	[2]
Imidazo[1,2‐α]pyridine	DMF/PBS	N/A	30	2025	[37]
Guaiazulene	DMSO/water	N/A	1720	2025	[38]
Carbazole	MeCN	2 mins	1.42		This work

#### 3.2.5 Interference Studies

**FIGURE 9:**
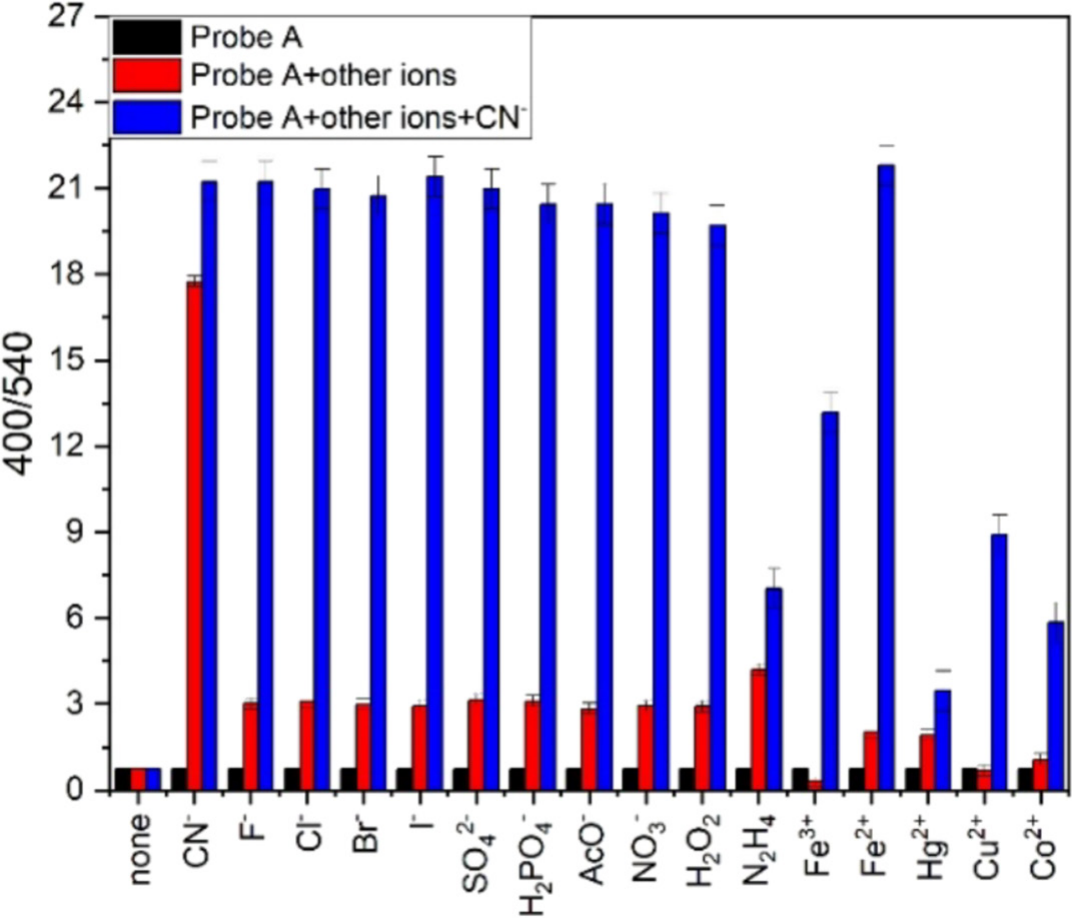
The I_400_/I_540_ ratio; probe **A** (black bars), probe **A** + competing analytes (red bars), probe **A**+competing analytes+CN^−^ (blue bars).

Sensing ability of probe **A** towards CN^−^ was also tested in the presence of various competing analytes. For this, a 30 μL of competing analyte (100 μM) was added to probe **A** (30 μM). Then, the emission of the mixture was recorded and the I_400_/I_540_ ratio was plotted for each competing analyte. The I_400_/I_540_ ratio increased 18‐fold only with the addition of CN^−^ (Figure 9, red bars). To each of the above mixtures, a 30 μL of CN^−^ (100 μM) was added to investigate the interference of competing analytes. Then, the emission of the mixture was recorded again and the I_400_/I_540_ ratio was re‐plotted for each analyte. In the presence of most competing analytes, the addition of CN^−^ increased the I_400_/I_540_ ratio 21‐fold except for N_2_H_4_, Fe^3+^, Hg^2+^, Cu^2+^ and Co^2+^ (Figure 9, blue bars). These results show that probe **A** can be used as a CN^−^ fluorescence sensor in the presence of most competing analytes.

#### 3.2.6 Proposed Sensing Mechanism

**SCHEME 1:**
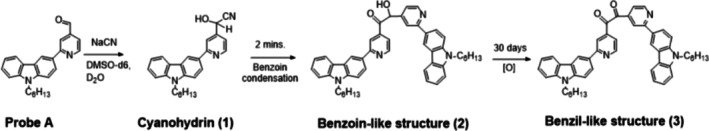
Proposed sensing mechanism of probe **A** for the detection of CN^−^.

Since CN^−^ is a strong nucleophile, probe **A** quickly reacts with CN^−^ to yield cyanohydrin (**1**). Surprisingly, cyanohydrin (**1**) further gives benzoin condensation to generate a benzoin‐like structure (**2**). Structures of probe **A**, cyanohydrin (**1**) and benzoin‐like structure (**2**) were confirmed by HRMS (see Supporting Information, Figure S1‐S3). Formation of benzoin‐like structure (**2**) can also be seen from ^1^H‐NMR spectra in which the aromatic region collapses to give a complex spectrum (see Figure S4). Upon long standing, the benzoin‐like structure (**2**) was also converted into the benzil‐like structure (**3**). Possible CN^−^ detection mechanism of probe **A** is depicted in Scheme 1.

**FIGURE 10:**
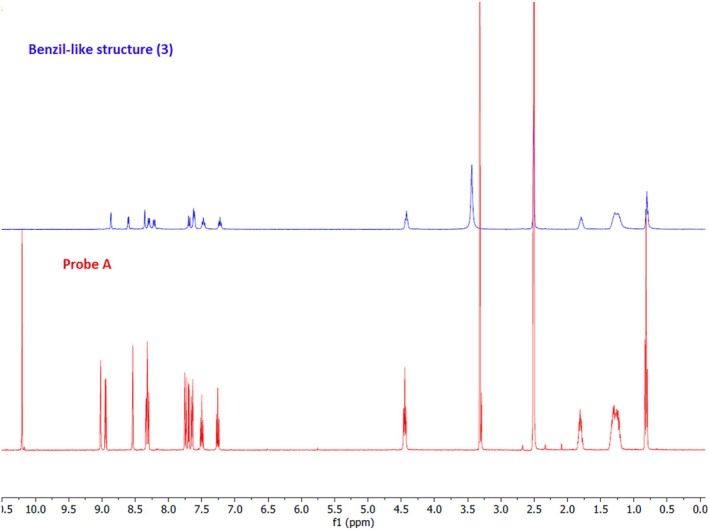
The ^1^H‐NMR spectra of probe **A** (red line) in DMSO‐*d6* and probe **A** + CN^−^ (blue line), indicating formation of benzil‐like structure (**3**) in DMSO‐*d6* + D_2_O.

The formation of **3** was confirmed by ^1^H‐NMR (Figure 10). After CN^−^ addition, in ^1^H‐NMR, an aldehydic proton peak at 10.21 ppm disappeared and a new peak at approximately 6.02 ppm for the hydrogen of cyanohydrin (**1**) was expected; however, it was not observed, indicating cyanohydrin (**1**) gives further reactions, as proved by HRMS.

Although only a few intramolecular benzoin condensation reactions in sensor studies have been reported in the literature [40, 41, 42], there is no report for intermolecular benzoin condensation in CN^−^ detection.

**FIGURE 11:**
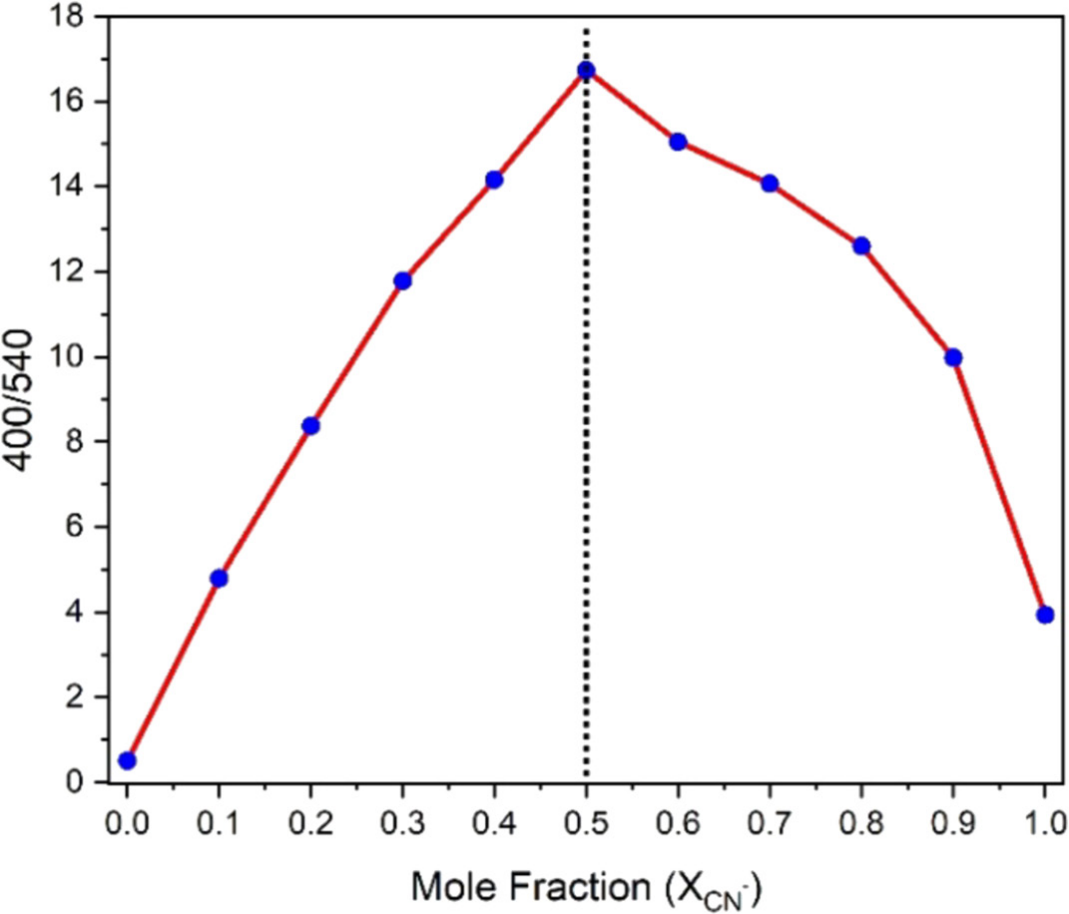
Job's plot between probe **A** and CN^−^.

The stoichiometric ratio between probe **A** and CN^−^ was also determined by Job's plot. Firstly, the solutions of probe **A** in MeCN and CN^−^ in deionized water were prepared at a concentration of 100 μM with a total volume of 1000 μL. These two solutions, with a total volume of 1000 μL, were mixed in different volumes of probe **A** and CN^−^, respectively, from 1000–0, 900–100, 800–200, 700–300, 600–400, 500–500, 400–600, 300–700, 200–800, 100–900, and to 0–1000. In this process, the mole fraction of CN^−^ was changed from 0, 0.1, 0.2, 0.3, 0.4, 0.5, 0.6, 0.7, 0.8, 0.9, and to 1. A 2000 μL of MeCN was added to each mixture. After that, the change in I_400_/I_540_ versus the mole fraction was plotted, as seen in Figure 11. From this graph, the mole fraction of CN^−^ was found to be 0.5, the point where I_400_/I_540_ is maximum. This value shows that probe **A** binds to CN^−^ in a 1:1 ratio.

**FIGURE 12:**
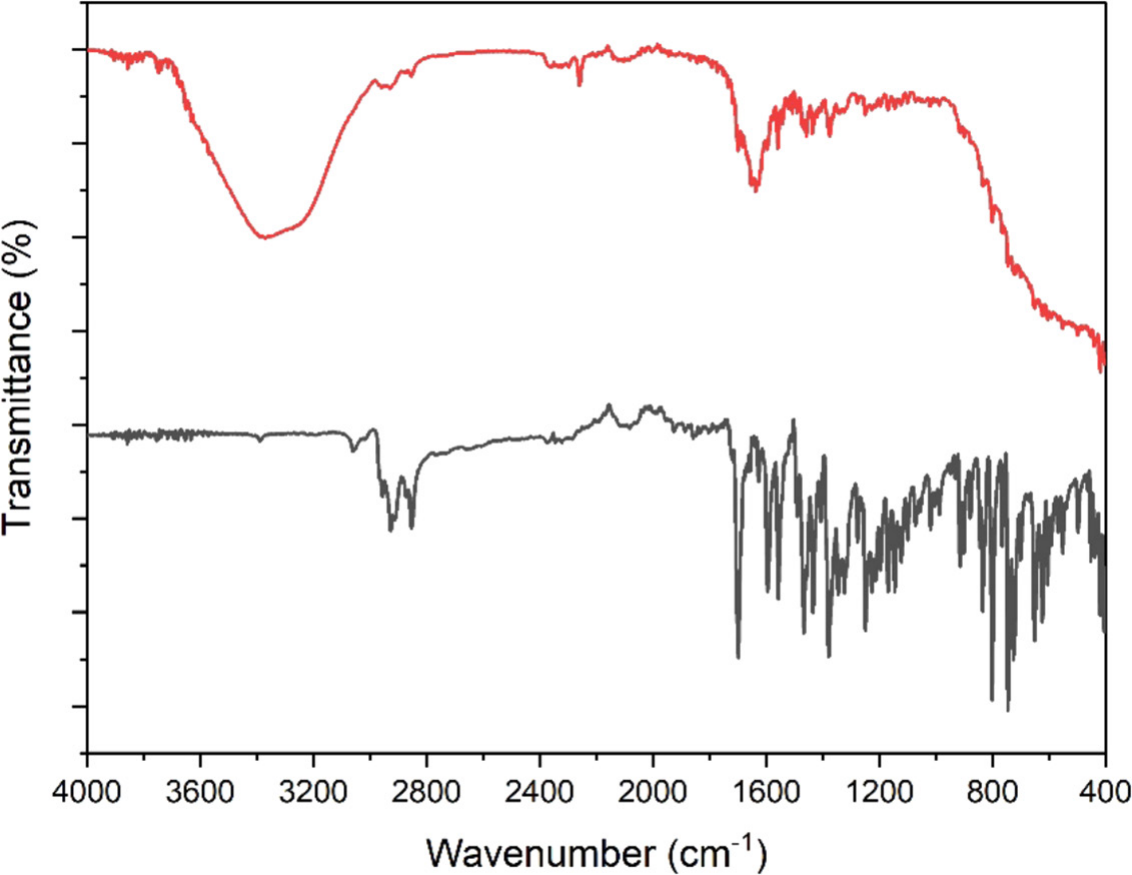
FTIR‐ATR spectra of probe **A** (black line) and probe **A** + CN^−^ (red line).

Besides Job's plot and ^1^H‐NMR analysis, the FT‐IR measurements of probe **A** in MeCN without and with the addition of CN^−^ in an aqueous solution were also carried out by using the ATR technique to find the sensing mechanism of probe **A** with CN^−^, as depicted in Figure 12.

The FT‐IR spectra of probe **A** in the solid state (Figure 12, black line) have a C=O stretching vibration peak at 1697 cm^−1^. Upon the addition of a CN^−^ solution (Figure 12, red line), the C=O stretching vibration peak at 1697 cm^−1^ disappeared and a new broad band at 3350 cm^−1^ appeared, indicating the formation of cyanohydrin (**1**).

#### 3.2.7 Practical Experiments in Real‐World Waters

To explore the applicability of probe **A** at three different real‐world water samples, the spike and recovery method was used. For this, first, 30 μL of each real water sample was separately added to probe **A** solutions in MeCN (30 μM, 2.7 mL). Each mixture was continuously spiked with different volumes of CN^−^. After waiting for the interaction between probe **A** and CN^−^, the emission of each mixture was measured three times. The I_400_/I_540_ ratio was plotted versus the added [CN^−^]. For each real‐world water sample, linear best fit equations were determined from the linear best fit lines. For probe **A**, experimental [CN^−^] (**e**) was found by using the I_400_/I_540_ ratio in the spiked mixture. In the blank mixture, [CN^−^] (**f**) was zero. Added [CN^−^] (**g**) was known. Hence, the recovery (%) was calculated by using [(**e**‐**f**)/**g**] equation. Also, the relative standard deviation (RSD) was calculated by using [100*(**s**/|**x̄**|)] equation. In there, (**s**) is the standard deviation of (**e**) and x̄ is the mean value of (**e**). The results of these experiments are summarized in Table 3. As shown in Table 3, depending on the real‐world water sample used, the obtained recovery (%) values are between 100.26% and 106.43%, with the maximum RSD value being less than 4%. Since the RSD data is below 10%, the measurements indicate probe **A** is largely accurate. Moreover, these results therefore showed that probe **A** can be used effectively to detect CN^−^ in real‐world water samples.

**TABLE 3:** Determination of the [CN^−^] in real‐world water samples.

Samples	CN^−^ added (μM)	CN^−^ found (μM)	Recovery (%)	RSD (%)
Probe A + tap water 1	60.00	60.61	101.02	0.79
Probe A + tap water 2	80.00	80.52	100.65	1.65
Probe A + tap water 3	90.00	93.04	103.38	2.39
Probe A + lake water 1	40.00	40.10	100.26	3.95
Probe A + lake water 2	60.00	61.13	101.90	0.65
Probe A + lake water 3	80.00	81.35	101.69	0.60
Probe A + river water 1	20.00	20.18	100.90	1.96
Probe A + river water 2	40.00	42.57	106.43	1.49
Probe A + river water 3	60.00	61.15	101.92	2.66

## 4 Conclusion

For the first time, in this work, probe **A** detects CN^−^ via a benzoin condensation reaction. Probe **A** is thus a novel carbazole‐based ratiometric fluorescence sensor. The sensing mechanism was fully investigated by PL, Job's plot, HRMS, and ^1^H‐NMR techniques. Probe **A** showed the lowest LOD value (1.42 nM) in the detection of CN^−^, which was well below the maximum level of CN^−^ (1.9 μM) set by WHO. Therefore, probe **A** can be a promising candidate for the detection of CN^−^.

## Supporting information


**Figure S1.** HRMS spectrum of probe **A**.
**Figure S2.** HRMS spectrum of cyanohydrin (**1**).
**Figure S3.** HRMS spectrum of benzoin‐like structure (**2**).
**Figure S4.** The ^1^H‐NMR spectra of probe **A** (red line) in DMSO‐*d6* and probe **A** + CN^−^ (blue line), indicating formation of benzoin‐like structures (**2**) in DMSO‐*d6* + D_2_O.

## Data Availability

The data that support the findings of this study are available from the corresponding author upon reasonable request.
