# Supplementary material for: 2‐(N‐Hexylcarbazole‐3′‐yl)‐4‐pyridinealdehyde: Cyanide Detection via Benzoin Condensation
Source: Luminescence. 2025 Aug 1;40(8):e70274. doi: 10.1002/bio.70274 (PMC12317201; doi:10.1002/bio.70274)
Supplement: Supplementary file 1 — Figure S1. HRMS spectrum of probe A. Figure S2. HRMS spectrum of cyanohydrin (1). Figure S3. HRMS spectrum of benzoin‐like structure (2). Figure S4. The 1H‐NMR spectra of probe A (red line) in DMSO‐d6 and probe A + CN− (blue line), indicating formation of benzoin‐like structures (2) in DMSO‐d6 + D2O. [file BIO-40-e70274-s001.docx]

**Supplementary Figure Captions**

**Fig. S1.** HRMS spectrum of probe **A**.

**Fig. S2.** HRMS spectrum of Cyanohydrin **(1)**.

**Fig. S3.** HRMS spectrum of Benzoin-like structure (**2**).



**Fig. S4.** The ^1^H-NMR spectra of probe **A** (red line) in DMSO-*d6* and probe **A**+CN^-^ (blue line), indicating formation of benzoin-like structures (**2**) in DMSO-*d6*+D_2_O.


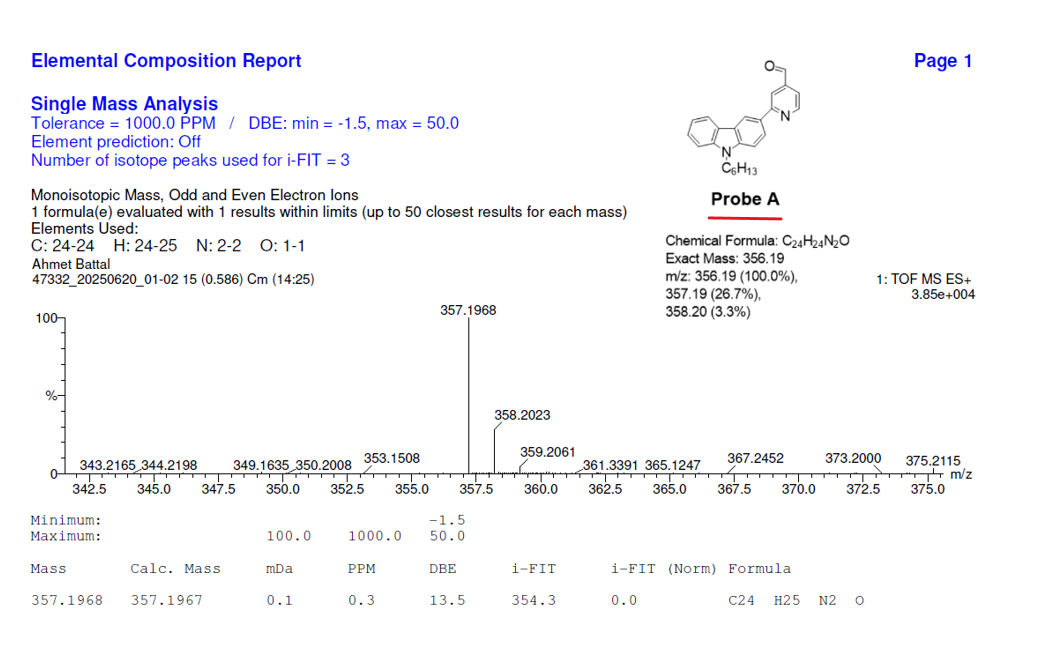



**Fig. S1.** HRMS spectrum of probe **A**.


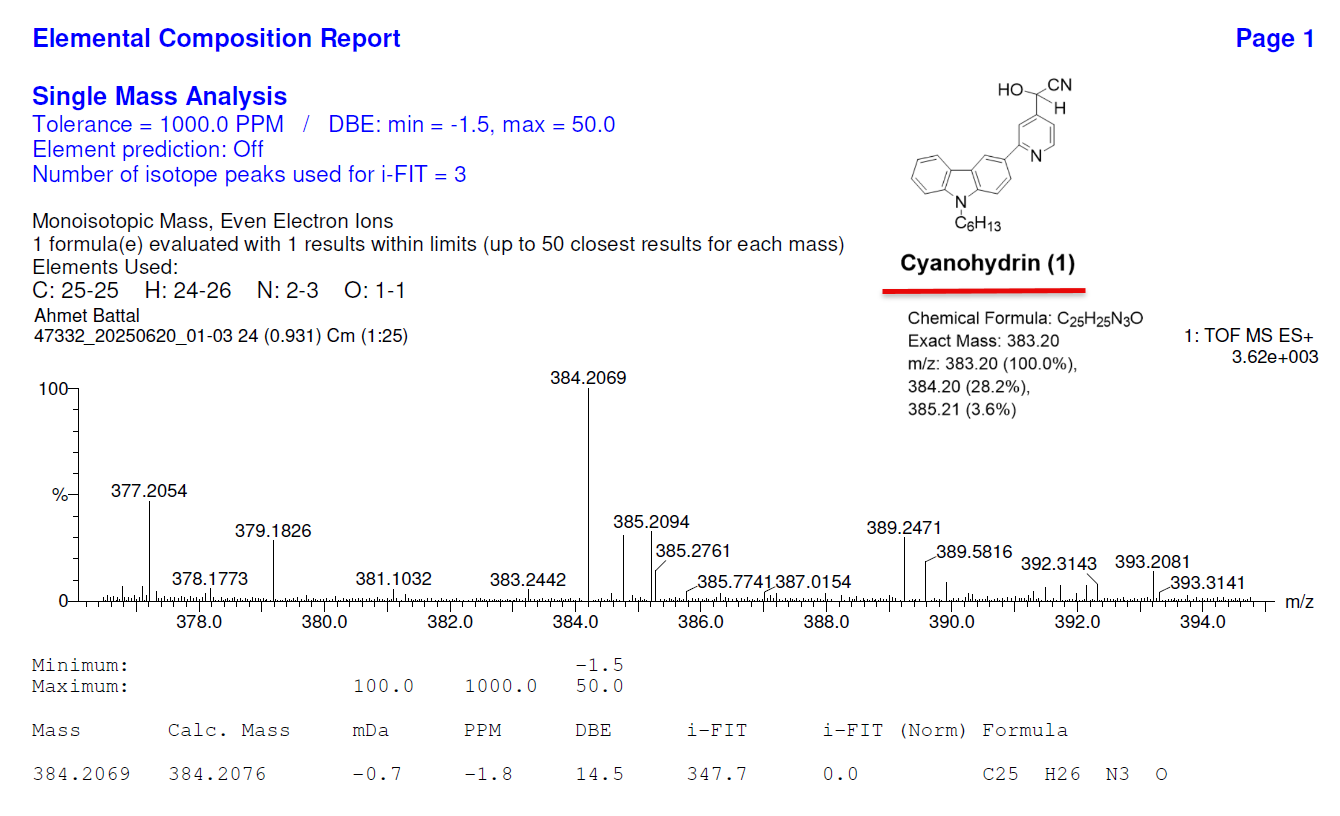



**Fig. S2.** HRMS spectrum of Cyanohydrin **(1)**.


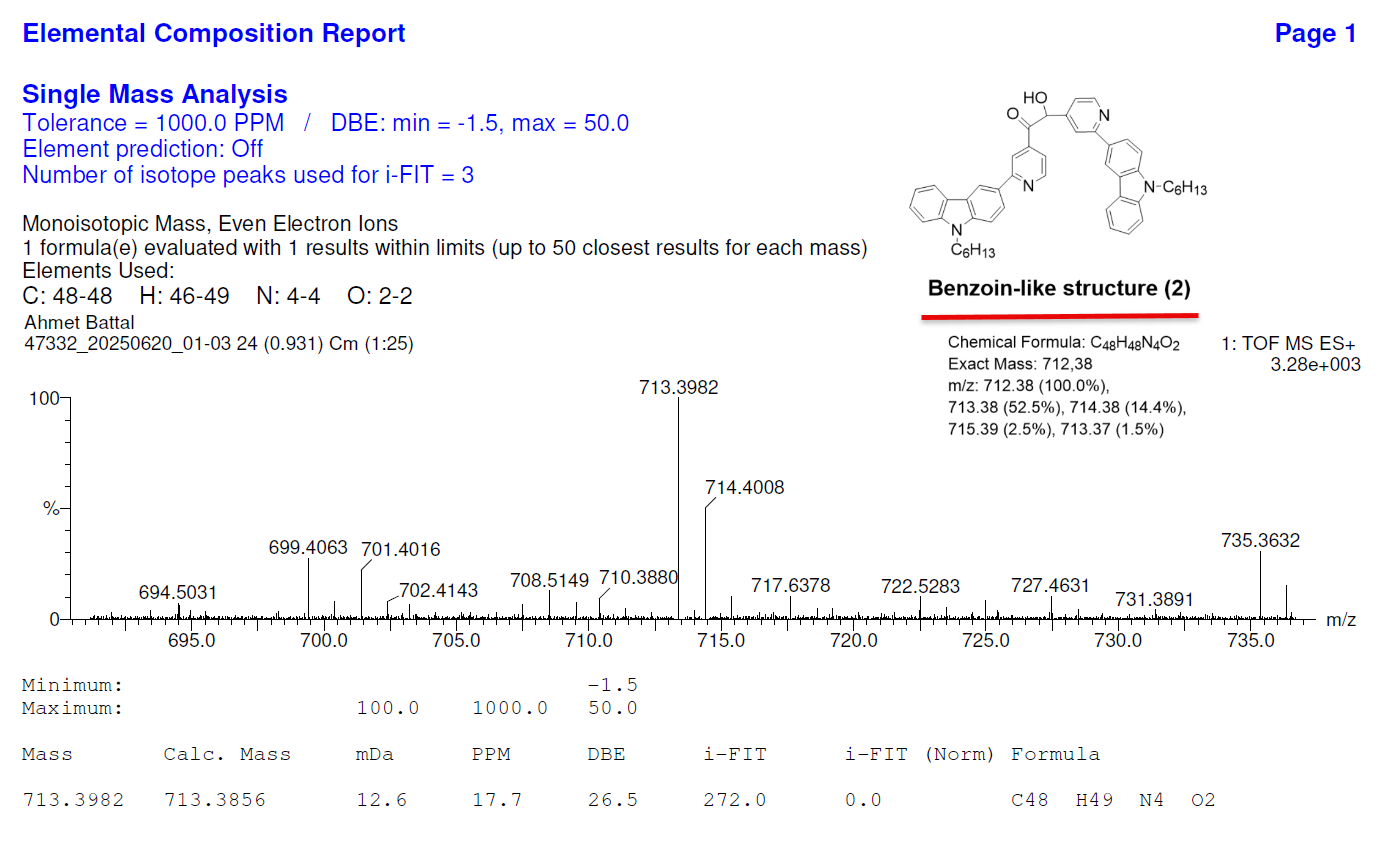


**Fig. S3.** HRMS spectrum of Benzoin-like structure (**2**).



**2- NMR spectra**


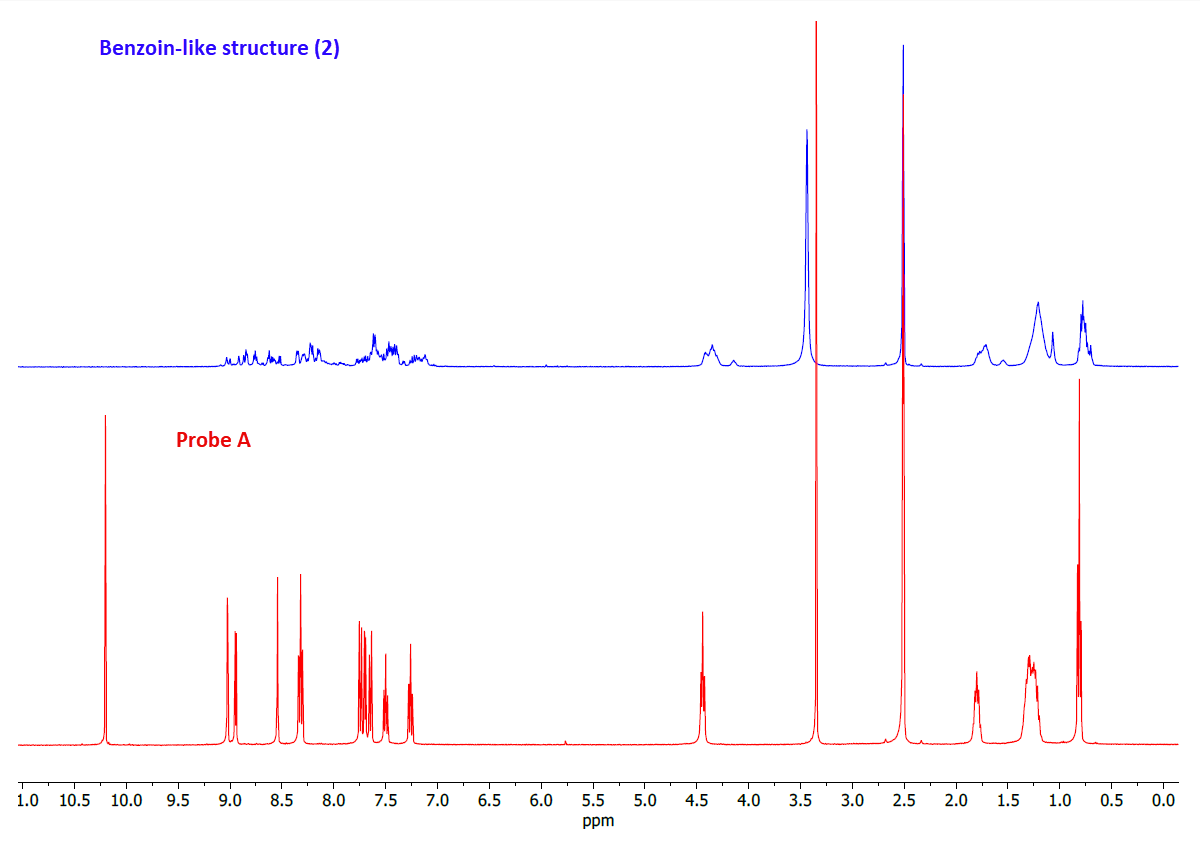


**Fig. S4.** The ^1^H-NMR spectra of probe **A** (red line) in DMSO-*d6* and probe **A**+CN^-^ (blue line), indicating formation of benzoin-like structures (**2**) in DMSO-*d6*+D_2_O.
